# Techno-economic assessment for plant seed-based production of Thaumatin II: a natural high intensity protein sweetener

**DOI:** 10.3389/fnut.2026.1868916

**Published:** 2026-07-23

**Authors:** Varun Gore, Daniel Tusé, Anatoli Giritch, Somen Nandi, Karen A. McDonald

**Affiliations:** 1 Department of Chemical Engineering, University of California, Davis, Davis, CA, United States; 2 DT/Consulting Group, Sacramento, CA, United States; 3 Nomad Bioscience GmbH, Halle (Saale), Germany; 4 Global HealthShare® Initiative, University of California, Davis, Davis, CA, United States

**Keywords:** environmental impact, natural protein sweetener, seed-based production, techno-economic modeling and analysis, Thaumatin II

## Abstract

**Introduction:**

A global rise in population and excessive consumption of dietary sugars present major environmental and public health challenges. The World Health Organization (WHO) recommends limiting free sugar consumption to less than 5% of total caloric intake for maximum health benefits. Substituting added sugar with low- or no-calorie sweeteners offers a viable alternative to achieve this target. Thaumatin II is a natural, non-caloric, high-intensity sweet-tasting protein, traditionally extracted as a mixture of up to 5 thaumatins from the fruit of *Thaumatococcus daniellii*, a plant native to West Africa, that can now be produced recombinantly in the seeds of temperate plants. Thaumatin II exhibits exceptional sweetness intensity (10,000–13,000 times that of sucrose, w/w) with minimal off-flavor characteristics.

**Methods:**

This study presents a techno-economic analysis for the large-scale recombinant production of Thaumatin II using transgenic *Nicotiana tabacum* seeds as a biomanufacturing platform. We also examine the impact of product purity on production cost and present a process mass intensity (PMI) evaluation and environmental, health, and safety (EHS) assessment to characterize overall process sustainability and environmental impact.

**Results:**

While the market benchmark price for the commercial extract (Talin^®^) is $7,000/kg, the analysis of the recombinant process projects a unit production cost of $766/kg for Thaumatin II with 95% purity (Thaumatin II/total soluble protein) and 5% moisture content, for a facility capable of producing 50 metric tons (MT) of Thaumatin II annually.

**Discussion:**

This study indicates that production of Thaumatin II in bioengineered plants can deliver significant economic and environmental advantages compared to traditional sweeteners and *T. daniellii* extraction methods, supporting the global effort to reduce added sugar consumption through sustainable, natural sweetener alternatives.

## 1 Introduction

This study is a techno-economic analysis of a novel biomanufacturing process to sustainably produce the sweet protein Thaumatin II for use in the full or partial replacement of sugar and other sweeteners. Excessive consumption of sugar is seen as a prime cause of non-communicable conditions such as unhealthy weight gain, obesity, cardiovascular disease and dental caries. Multinational programs have been implemented to prevent and control unhealthy weight gain and the proliferation of diet-related chronic diseases. In 2015, the WHO issued guidelines for reducing the risk of non-communicable diseases, principally weight gain and tooth decay. The guidelines recommended that adults and children consume less than 10% of their total daily calories from free sugars (i.e., caloric sweeteners including sucrose, glucose, high-fructose corn syrup (HFCS), etc.), and further suggested that additional health benefits would derive if free sugar consumption was less than 5% of total daily calories (1). Consumption of food and beverages containing non-nutritive sweeteners to replace all or some of the added sugar could help consumers achieve the WHO recommendations for sugar reduction. Importantly, non-sugar sweeteners can be beneficial for those with diabetes, as promoted in recent guidance from the American Diabetes Association (2).

Approximately two dozen non-sugar sweeteners are available for sugar replacement. Each could be used singly or in combination with sugar and/or other sweeteners depending on the intended application. Currently there are three main categories of sweeteners approved for reduced-calorie applications: (1) low- and no-calorie sweeteners (LNCS, including acesulfame-potassium (ace-K), advantame, alitame, aspartame, aspartame-acesulfame salt, cyclamate, monk fruit extract (also known as Luo Han Guo), neotame, saccharin, steviol glycosides, sucralose, and thaumatin); (2) reduced-calorie sweeteners, more commonly known as sugar alcohols or polyols (erythritol, hydrogenated starch hydrolysates (polyglycitol syrup), isomalt, maltitol, maltitol syrup, mannitol, sorbitol, sorbitol syrup, and xylitol), and (3) rare sugars (allulose, isomaltulose, and tagatose).

These categories of sweeteners, and in particular LNCS, are among the most studied ingredients of food and beverages and their safety has been affirmed by multiple international scientific and regulatory authorities, including the Joint Food and Agriculture Organization and World Health Organization (FAO/WHO) Expert Committee on Food Additives (JECFA), the European Food Safety Authority (EFSA), the United States (US) Food and Drug Administration (FDA), Food Standards Australia and New Zealand (FSANZ), Health Canada, and others (3). As the demand for reduced-sugar and reduced-calorie products continues to grow, LNCS, in particular, are expected to play a more significant role in sugar-reduction and sugar-replacement efforts.

In addition to the positive impacts on health of reduced sugar diets, reduced sugar use would be expected to lead to reduced sugar production, a practice that is associated with negative environmental impacts due to deforestation, eutrophication, soil erosion and loss of soil quality, especially in subtropical and tropical environments (4). Global sugar production volumes in 2024–2025 reached nearly 181 million metric tons (MT) (5). The irrigation practices for growing crops to produce sugars (e.g., sugarcane, sugar beet, corn), and sugarcane in particular, are water intensive. About 80% of the world’s sugar comes from sugarcane (6). The World Wildlife Fund (4) estimated that sugarcane covers more than 26 million hectares (65 million acres) of land worldwide, and that a dozen countries use at least 25% of their farmland to grow it. Approximately 1,500–3,000 liters of water are required to produce 1 kg of refined sucrose from sugarcane (~180–360 gal/lb), which amounts to about 6–13 liters of water per teaspoon of sugar (1.5–3.5 gal/tsp) (4).

In the US, sucrose from cane and beet and HFCS from corn together account for 90% of the sweeteners consumed (7). As the world’s population increases, satisfying demand for carbohydrate (caloric) sweeteners using current practices may be challenging, especially when considering the growing consumer preference for sustainably produced food ingredients (8). Hence, adoption of sweeteners whose production minimizes such environmental consequences would offer a welcome change with respect to both health and sustainability.

The approximately two dozen non-nutritive sweeteners available today are produced using a variety of processes; some are made synthetically, some semi-synthetically, while others are extracted from natural sources and purified for integration into food and beverages. Despite their safety record, legislation to limit the use of synthetic non-sugar sweeteners in children’s diets has been proposed due to concerns about their long-term health effects, as some studies suggest non-sugar sweeteners may not help with weight control and could increase the risk of type 2 diabetes and cardiovascular disease, impact the preference for sweet tastes, and potentially cause harm in early development, although such concerns have not always been supported by scientific evidence (9). Natural sweeteners such as stevia, monk fruit and thaumatin have largely escaped such concerns because of their digestibility and lack of glycemic and gastrointestinal effects. In the case of thaumatin, these same attributes are further complemented by the protein mixture’s high potency, which allows for its application to food and beverages at very low levels compared with sugar and lower-potency sweeteners, further increasing its safety margin.

Food and beverage formulators strive to reduce added sugars in their products without changing the sweetness and taste profile of the food and beverages, mainly by increasingly relying on natural high-intensity sweeteners; that is, sweeteners that induce a potent sweetness sensation at very low concentrations. For successful large-scale adoption as a replacement for sugar, an ideal natural sweetener should be safe, able to maintain the sweetness that the consumer is used to without significantly changing the taste profile of a product, replace or complement both sugars and other sweeteners, offer a better sweetness cost in use, and benefit from supply security to make its adoption attractive to high-volume users such as soft drink and sweetened beverage companies. In this study, we analyze a natural high-intensity protein sweetener (Thaumatin II) whose sweetness functionality and manufacturing method could meet these key criteria.

The thaumatin protein mixture Talin® (introduced by Tate and Lyle in the 1970s; designated with a small “t” in this study to differentiate it from the single protein Thaumatin II), was one of the earliest natural non-caloric high intensity products on the market. Thaumatin has been approved for use as a food additive in multiple countries, typically as a non-caloric sweetener and/or taste modifier (e.g., as a sweetener in the EU, Japan and Israel, and as a taste modifier in the US). The extracted thaumatin protein mixture is 2,000-3,500-times sweeter than sucrose on a weight basis and is thus the sweetest natural product. However, thaumatin currently serves a niche market as a taste modifier, and not as a sweetener, because of its limited supply (industry estimates range from ~1.5 MT/yr). (10) to >130 MT/yr., with 65 MT/yr. as a midpoint (11) and variable supply security of the natural source (arils of the fruits of *Thaumatococcus daniellii* (Benth) plants, a shrub undergrowth in the forests of West Africa). Attempts to establish large-scale commercial plantations of *T. daniellii* in Africa and other areas to supply the sweetener market have not succeeded, in no small part due to the peculiar growing requirements of the shade-loving plant (12). In addition, the extracted thaumatin protein blend, consisting of the thaumatin proteins plus host and process impurities, suffers slightly from undesirable taste attributes including a lingering aftertaste and liquorice-like off notes (13).

To overcome these limitations in supply, sensory attributes and range of applications, interest developed in producing Thaumatin II, a 22 kDa MW protein with a pI of 11.5–12.5 and one of the main components of thaumatin mixtures, using recombinant technology. Recombinant Thaumatin II can now be produced in plants that can be grown in any temperate region via a scalable process and thus its supply is not constrained by the agronomic limitations of the naturally harvested product. In recent years, Thaumatin II has been produced recombinantly using both transient and stable gene expression in *Nicotiana* species of plants as well as in edible crops; processes that we modeled and analyzed previously (14–16). Importantly, plant-produced recombinant Thaumatin II has been categorized by the FDA as Generally Recognized as Safe (GRAS) for use as both a sweetener and flavor modifier (17–19) and by the Flavor and Extract Manufacturers Association (FEMA) as GRAS for use as a flavor with modifying properties (20).

Replacing a significant volume of dietary sugar (even partially) with LNCS can only be realized by the availability of scalable and cost-effective manufacturing processes that are also environmentally benign. Such processes exist for other non-nutritive sweeteners, but not for thaumatin at the scales needed to satisfy the sweetener market (as opposed to the smaller flavor modifier market served today due to constrained supply via the extraction route).

This study is a techno-economic analysis of the sweet protein Thaumatin II recombinantly expressed in transgenic tobacco seeds. When formulated to replace 35–50% of total sweetness from added sugar, Thaumatin II exhibits exceptional sweetness intensity (10,000–13,000 times that of sucrose, w/w) with minimal off-flavor characteristics (18). The large-scale manufacturing facility that is modeled is capable of producing 50 metric tons of Thaumatin II annually at 95% purity and with 5% moisture content. The single facility can supply >75% of the current estimated average global volume of extracted thaumatin (avg. ~65 MT/yr) and can be replicated at multiple sites to accommodate future demand for the natural sweetener. We also examine the impact of production volume and product purity on production cost and conduct a process mass intensity (PMI) evaluation and an environmental, health, and safety (EHS) assessment to define overall process sustainability and environmental impact of the designed facility.

## 2 Materials and methods

### 2.1 Process modeling and economics

Process simulations and economic analyses were performed using SuperPro Designer® Version 14 Build 1 (Intelligen, Inc., Scotch Plains, NJ, USA; http://www.intelligen.com). This software allows modeling of various widely used process unit operations, including equipment sizing, performing mass and energy balances, batch scheduling, and determining capital expenditure (CAPEX), annual operating expenditure (OPEX), and cost of goods sold (COGS). The model inputs and assumptions are process and economic parameters obtained from working process knowledge, laboratory and pilot-scale data from the authors’ laboratories and bioprocessing facilities, published data from the literature, and SuperPro default values.

The base case SuperPro Designer® file is publicly available at https://mcdonald-nandi.ech.ucdavis.edu/tools/techno-economics/. A free trial download of SuperPro Designer (https://www.intelligen.com/download/) can be used to view the models, run the simulations, and change process parameters/assumptions (although changes cannot be saved).

### 2.2 Model assumptions and design

The base case scenario assumes an annual production capacity of 50 MT of Thaumatin II, produced over 293 batches per year. The base case production level of 50 MT/yr. of Thaumatin II was selected based on the reported ranges of thaumatin production from ~1.5 MT/yr. (10) to >130 MT/yr., with a mid-point around 65 MT/yr. (11); our target was selected to be around 76% of the midpoint value. However, a scenario analysis is performed to evaluate the economics for production facilities ranging from 25 MT/yr. to 150 MT/yr. The upstream production cost is derived from the cost of cultivating and harvesting *Nicotiana tabacum* seeds through open-field growth. The downstream processing batch time is 83.7 h, with a recipe cycle time (the time between the start of consecutive batches) of 26.8 h. Key process assumptions and their sources are summarized in Table 1.

**Table 1:** Key design assumptions and parameters for the downstream production facility.

Parameter	Value	Source
Annual production	50 MT	Assumption based on 75% of the midpoint for current annual thaumatin production; scenario analysis for 25 MT to 150 MT annual production is considered in Section 2.3
Thaumatin II content in seed	8 g/kg	Pilot scale experimental data
Total Soluble Protein (TSP)	320 g/kg	Pilot scale experimental data
Final Purity (Thaumatin II/TSP)	95%	Pilot scale experimental data
Downstream recovery	53.4%	Pilot scale experimental data
Annual Operating time	330 days	Assumption based on 90% of the maximum annual operating time with 10% of the time for scheduled and unscheduled down time
Batches per year	293	Calculated based on the annual operating time, process cycle time, and process batch time

**Figure 1:**
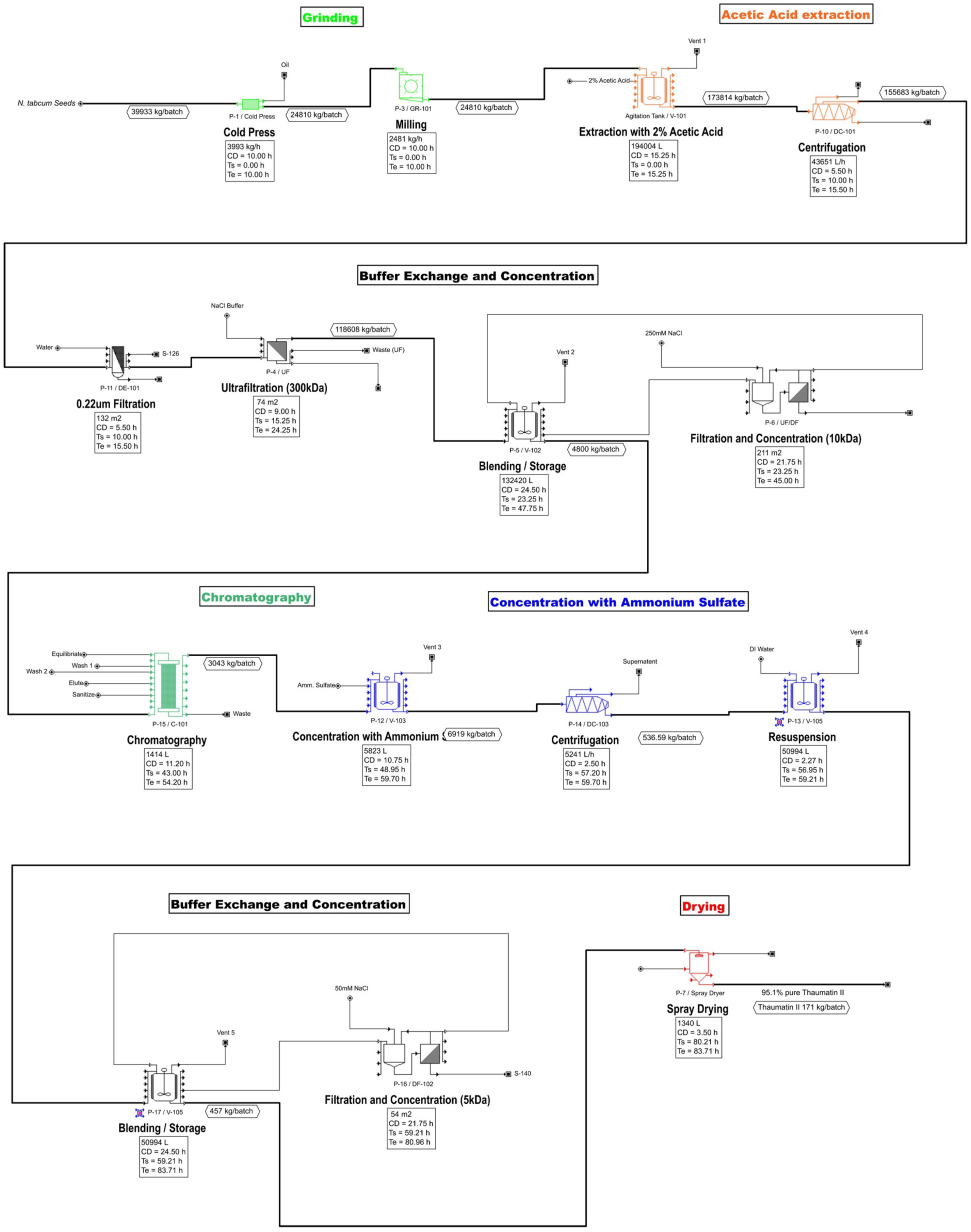
Process flow diagram of the downstream purification process facility. The facility integrates the following process flow: Cold press and grinding, acetic acid extraction, buffer exchange and concentration, chromatography, precipitation using ammonium sulfate, a second buffer exchange, and spray drying. Equipment P-12 (resuspension) and P-13 (blending/storage) are considered the same, as indicated by the small pink symbol (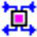) at the bottom left of the equipment. CD: cycle duration; T_s_: start time; T_e_: end time.

The process flow diagram for the downstream recovery facility is shown in Figure 1. The feed for each batch is 39,933 kg of *Nicotiana tabacum* transgenic seeds with a composition shown in Table 2. The facility is comprised of a cold press, holding tanks, decanter centrifuges, an ultrafiltration (UF) skid, an ultrafiltration/diafiltration (UF/DF) skid, a cation exchange chromatography column, and a spray dryer. The cold press is used to separate seed oil from the seed biomass. Proteins are extracted from *Nicotiana tabacum* seed biomass in an agitated tank containing 2% (v/v) acetic acid for 1.5 h. The resulting precipitated proteins and residual biomass are separated using a decanter centrifuge, and the clarified supernatant is passed through a 0.22 μm membrane followed by a 300 kDa UF membrane. The permeate is subsequently processed through a UF/DF skid with a 10 kDa membrane to achieve a 25-fold concentration and buffer exchange into a 250 mM NaCl solution. The retentate from the UF/DF step is loaded onto a cation exchange chromatography column containing Cytiva Capto S resin with an assumed dynamic binding capacity of 150 g Thaumatin II/L. This value is similar to the manufacturer’s stated dynamic binding capacity for lysozyme at 10% breakthrough (120 g/L) and was used to keep the process consistent with the process simulated by Kelada et al. (14). The eluate from the chromatography column is transferred to an agitation tank, where ammonium sulfate is added and the solution is incubated for 6.75 h to induce Thaumatin II precipitation. The precipitate collected from the decanter centrifuge is resuspended in deionized water and subsequently subjected to UF/DF using a 5 kDa membrane for buffer exchange and concentration. This step removes residual ammonium sulfate and concentrates the target protein prior to drying. The final retentate is spray-dried to reduce moisture content, yielding a product with 95% solids. Additional model parameters can be found in the Supplementary materials for individual unit product recoveries (Supplementary Table S1), direct fixed capital factors (Supplementary Table S2), working capital parameters (Supplementary Table S3) and other operating cost parameters (Supplementary Table S4).

**Table 2:** *N. tabacum* seed composition (44–46).

Component	Amount
Protein	18–41%
Carbohydrate	3.79–4.03%
Fat	36.31–39.34%
Fiber	3.7–21.8%
Polyphenols	288–357 mg GAE/100 g

All values are reported on a dry weight basis. GAE is gallic acid equivalent.

The base-case model assumes a production cost of $2/kg of *N. tabacum* seed. This seed cost was derived empirically from our multi-year experimental field studies and includes labor and material costs for upstream agricultural practices (land preparation, crop cultivation, irrigation and maintenance, seed harvest and clean-up), plus regulatory costs for open-field cultivation and segregation of a transgenic crop at pilot (non-commercial) scale. A sensitivity analysis was performed to evaluate the impact of seed cost on the final production cost of Thaumatin II.

### 2.3 Scenario analysis

Production facilities with annual throughputs ranging from 25 to 150 MT/yr., in 25 MT/yr. increments, were designed to evaluate the impact of scale on production economics. All facilities were configured to operate for 293 batches per year and to produce Thaumatin II at 95% purity with a final solids content of 95%. The amount of Thaumatin II in the final dry product depends on how the facility operations are configured. To illustrate, the production facility was modified by selectively removing unit operations to evaluate the manufacture of Thaumatin II at different purity levels. In Scenario 1, the chromatography section shown in Figure 1 was omitted, while all other unit operations were retained. This configuration was designed to produce 50 MT of Thaumatin II annually across 293 batches, achieving a downstream recovery of 60.5%. The resulting final product stream (104.6 MT/yr) exhibited a purity of 52.5%. In Scenario 2, the ammonium sulfate concentration step shown in Figure 1 was removed, while all remaining unit operations were maintained. This facility configuration was designed to produce 50 MT of Thaumatin II annually over 321 batches, achieving a downstream recovery of 54.2%. The final product stream (58.3 MT/yr) achieved a purity of 92%.

### 2.4 Process mass intensity

Process Mass Intensity is a metric used to evaluate the resource efficiency of production processes. PMI is calculated as the total mass of water, raw materials, and consumables required (in kilograms) per kilogram of active product produced (21). This method has been used as a sustainability metric for a wide range of biomanufacturing processes ranging from peptide synthesis (22) to plant-made biopharmaceuticals (23). In this study, the mass inputs of acids and bases used for cleaning are included in the PMI calculations. All solutions are assumed to be prepared in-house; therefore, the mass of water used to prepare these solutions is incorporated into the water PMI. This analysis only includes the downstream processing of the seeds to produce purified Thaumatin II (Figure 1); resources for the upstream, agricultural production and harvesting of the seeds, are not included.

### 2.5 Environmental impact assessment

The Environment, Health, and Safety assessment of the process was conducted using the environmental evaluation method developed by Biwer and Heinzle (24). This method has been used in many studies to evaluate the impact of manufacturing processes on the environment as well as the health and safety of manufacturing personnel (25, 26). The method evaluates process inputs and outputs scaled to the quantity of product produced, referred to as the Mass Index (MI). The assessment considers the impacts on natural resource depletion, air quality, water and soil quality, hazards, and toxicity to humans, plants, and animals. The environmental significance of each material used as an input or generated as an output is represented by its Environmental Factor (EF). These factors are derived from fourteen impact categories, with each compound classified using the ABC semi-qualitative system: A (high negative impact), B (moderate impact), and C (low or negligible impact). The impact categories are further grouped into six broader impact groups: Resources, Gray Input, Component Risk, Organisms, Air, and Water/Soil. Environmental factors for inputs and outputs are derived from these impact groups. The Environmental Index (EI) of a component is determined by weighing its Mass Index (MI) with its Environmental Factor (EF). The total EI for the process is calculated by summing the EI values of all inputs and outputs, providing an indicator of the process’s overall environmental relevance. The CO_2 eq_ is calculated using the U. S. Energy Information Administration (27) as 1.05 kg CO_2_/kWh (coal), 0.44 kg CO_2_/kWh (natural gas), 1.12 kg CO_2_/kWh (petroleum), and 0.37 kg CO_2_/kWh (all energy sources). Similarly to the PMI, this analysis only includes the downstream processing of the seeds to produce purified Thaumatin II (Figure 1); EHS assessment for the upstream, agricultural production and harvesting of the seeds, is not included.

## 3 Results

### 3.1 Base case model economic analysis

The total capital expenditure for a proposed US-located facility is estimated at 26.3 million US dollars (USD). This includes equipment purchase costs of 6.6 million USD, other direct costs (piping, instrumentation, insulation, electrical, and auxiliary facilities) totaling 8.5 million USD, and indirect costs (engineering and construction) amounting to 4.7 million USD, contractor fees and contingencies estimated at 3 million USD, adding to 22.8 million USD as the direct fixed capital. Additional expenses for working capital are 2.4 million USD, and start-up and validation costs add up to 1.1 million USD.

**Figure 2:**
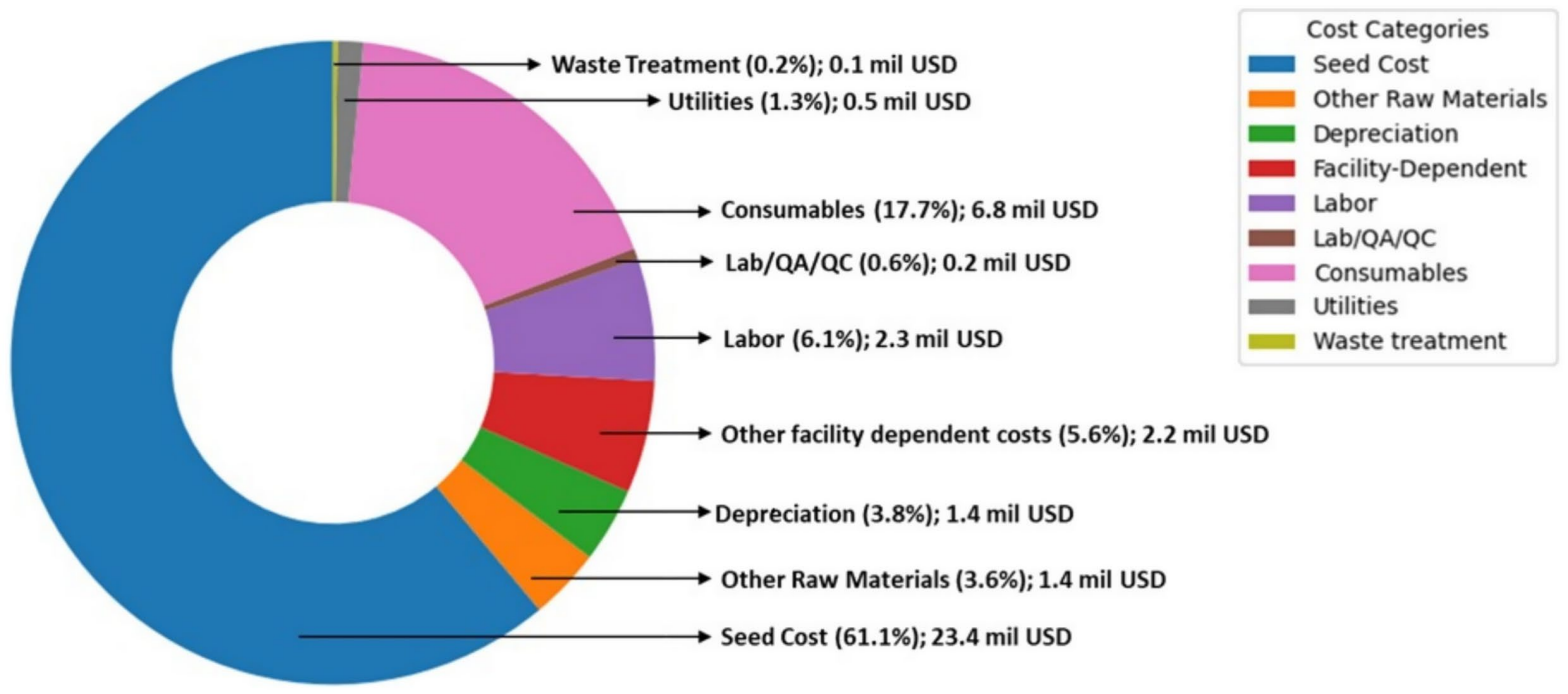
Downstream operating cost breakdown for the proposed base case seed-based Thaumatin II production facility.

The annual operational expenditure is estimated at 38.3 million USD (Figure 2) with major cost contributors including seed cost (23.4 million USD/yr., 61.1%.), consumables (6.8 million USD/yr., 17.7%), other facility-dependent costs such as maintenance, insurance, and local taxes (2.2 million USD/yr., 5.6%), depreciation (1.4 million USD/yr., 3.8%), labor (2.3 million USD/yr., 6.1%), and raw materials (excluding the seed cost) (1.4 million USD/yr., 3.6%). Laboratory quality control and assurance (0.2 million USD/yr., 0.6%), utilities (0.5 million USD/yr., 1.3%), and waste treatment and disposal (0.1 million USD/yr., 0.2%) constitute the remaining expenses. Notably, chromatography resin accounts for approximately 33.6% of the total downstream purification, reflecting the consumable cost associated with downstream purification.

**Figure 3:**
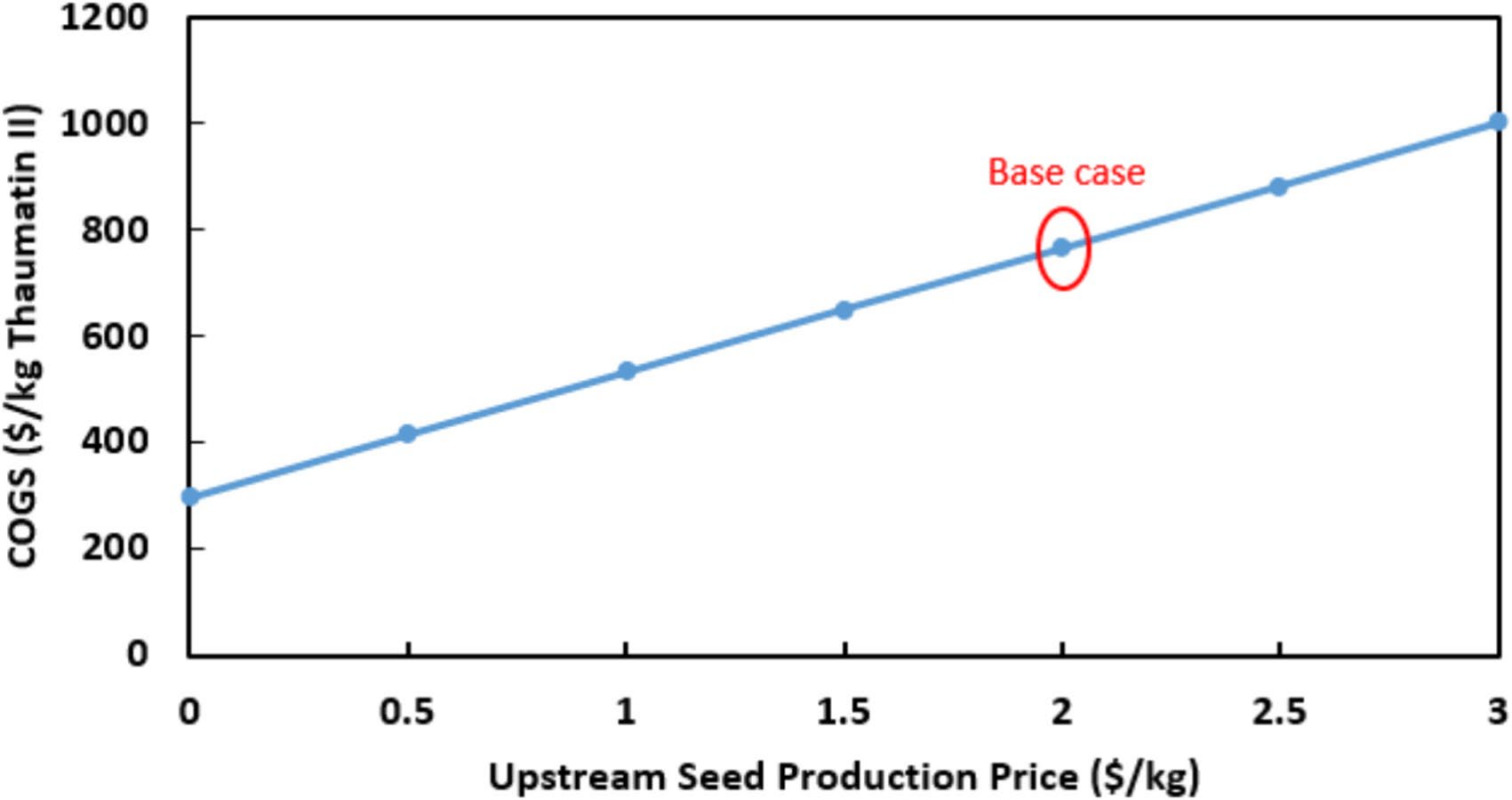
Variation of COGS for the production of Thaumatin II as a function of upstream seed production cost. The COGS when seed production cost is neglected (downstream purification cost only) is $298/kg Thaumatin II, and the COGS is $766/kg Thaumatin II if a seed production cost of $2/kg is assumed.

The estimated cost of goods sold for Thaumatin II production, including depreciation, is 766 USD/kg, and 723 USD/kg when depreciation is excluded. The sensitivity analysis (Figure 3) shows the cost of Thaumatin II increases linearly with an increase in seed production cost. Preliminary results we obtained from pilot-scale field studies indicate that the seed production cost is ~$2/kg, which was used as the base case scenario. The minimum selling price (MSP) at breakeven (defined as the selling price at which the internal rate of return (IRR) after a 25-year project life, 10-year straight-line depreciation, at a 40% tax rate is zero) is $747/kg, and the MSP to achieve 10% IRR is $814/kg. Table 3 shows a summary of the process economics for the seed-based Thaumatin II production facility.

**Table 3:** Summary of production facility economics.

Parameter	Base case
Annual Thaumatin II production (MT)	50
CAPEX (million USD)	26.3
Upstream seed production cost (USD/kg)	2
OPEX (million USD)	38.3
COGS with depreciation (USD/kg)	766
COGS without depreciation (USD/kg)	723
MSP (at breakeven) (USD/kg)	747
MSP (at 10% IRR) (USD/kg)	814

All USD values are based on the year 2026. CAPEX, Capital expenses; OPEX, Operating expenses; COGSm Cost of Goods Sold; MSPm Minimum selling price. The MSP is calculated for a facility with a 25-year lifespan, 10-year straight-line depreciation, and a 40% tax rate, at breakeven (0% IRR) and 10% IRR.

For comparison, the transgenic *N. tabacum* green leaf-based production processes we reported in Kelada et al. (2021) (14) yielded COGS (including depreciation) of $84.5/kg for upstream field-grown *N. tabacum*, $2,200/kg for upstream indoor-grown *Nicotiana benthamiana*, and $706/kg for downstream purification using chromatography. The total COGS for the field-grown *N. tabacum* platform was reported as $790.5/kg (i.e., $84.5 + $706). In contrast, the seed-based production process proposed in this study results in a total COGS of $766/kg. This represents a modest reduction in manufacturing cost compared to the field-grown *N. tabacum* system and a substantial reduction relative to the indoor-grown *Nicotiana benthamiana* green leaf platform. Although the seed-based and field-grown leaf-based processes exhibit comparable overall production costs, the seed-based platform offers important logistical and operational advantages. Seeds provide better stability and extended storage capability compared to fresh green leaf biomass, which is highly perishable and requires rapid downstream processing. These characteristics make the proposed seed-based process a more robust and commercially attractive production strategy.

### 3.2 Scenario analysis

#### 3.2.1 Variation in facility production size

**Figure 4:**
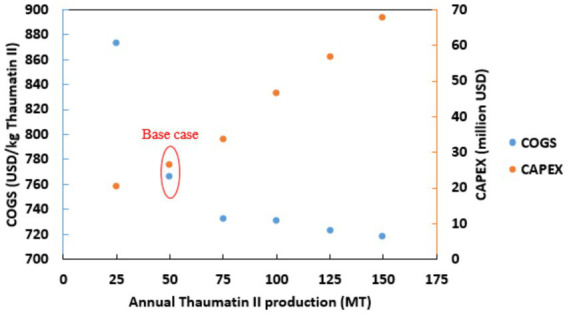
COGS and CAPEX as functions of facility throughput. All the facilities produce 95% pure Thaumatin II. COGS, Cost of Goods Sold; CAPEX, Capital expenses.

As shown in Figure 4, the COGS decreases with increasing facility throughput, indicating the impact of economies of scale. A steep reduction in COGS is observed when capacity increases from 25 to 50 MT/yr., however, further increases in throughput result in progressively smaller reductions in COGS, with the curve tapering beyond approximately 100 MT/yr. Capital expenditure exhibits an overall increasing trend with throughput. The relationship is approximately linear between 25 and 75 MT/yr. and again from 100 to 150 MT/yr. The deviation from linearity observed between 75 and 100 MT/yr. is attributable to the additional blending tanks required to accommodate the higher processing volume. Although additional reductions in COGS are achieved when scaling from 50 MT/yr. to 75 or 100 MT/yr., these savings are insufficient to offset the corresponding increase in capital investment. Consequently, an initial facility throughput of 50–75 MT/yr. represents a balanced starting point, with the flexibility to scale up or scale out in response to market demand and product requirements.

#### 3.2.2 Variation in product purity

Table 4 shows reduced CAPEX and OPEX for Scenarios 1 and 2 compared with the base case. As discussed in Section 3.1, chromatography resin is a major cost driver in downstream purification, which explains the lower OPEX observed for Scenario 1. The reduction in CAPEX arises from decreased equipment purchase costs, as well as lower direct and indirect costs and reduced working capital requirements. Although the production cost in Scenario 1 is ~24% lower than that of the base case, the resulting product has a final purity of 52.5%. This is lower than the purity of current, extracted thaumatin mixtures, which ranges from 70 to >90% (thaumatin protein/TSP) (28), underscoring the importance of the chromatography step in the process. Scenario 2 yields a more favorable outcome, with significant reductions in both CAPEX and OPEX while maintaining relatively high product purity, and similar downstream recovery. The observed cost savings justify the 2.5% reduction in purity relative to the base case, indicating that Scenario 2 is a viable process alternative.

**Table 4:** Summary of downstream purification facility economics when producing Thaumatin II at various purity levels.

Parameter	Base case	Scenario 1 (No chromatography)	Scenario 2 (No Ammonium sulfate concentration)
Purity (Thaumatin II/TSP)	95%	52.5%	92.50%
Annual production (MT)	50	50	50
CAPEX (million USD)	26.3	20.9	21.3
OPEX (million USD)	38.3	29.1	36.5
COGS with depreciation (seed cost at $2/kg) ($/kg)	766	583	729
MSP at breakeven ($/kg)	747	568	707
MSP at 10% IRR ($/kg)	814	622	757

### 3.3 Process mass intensity

**Figure 5:**
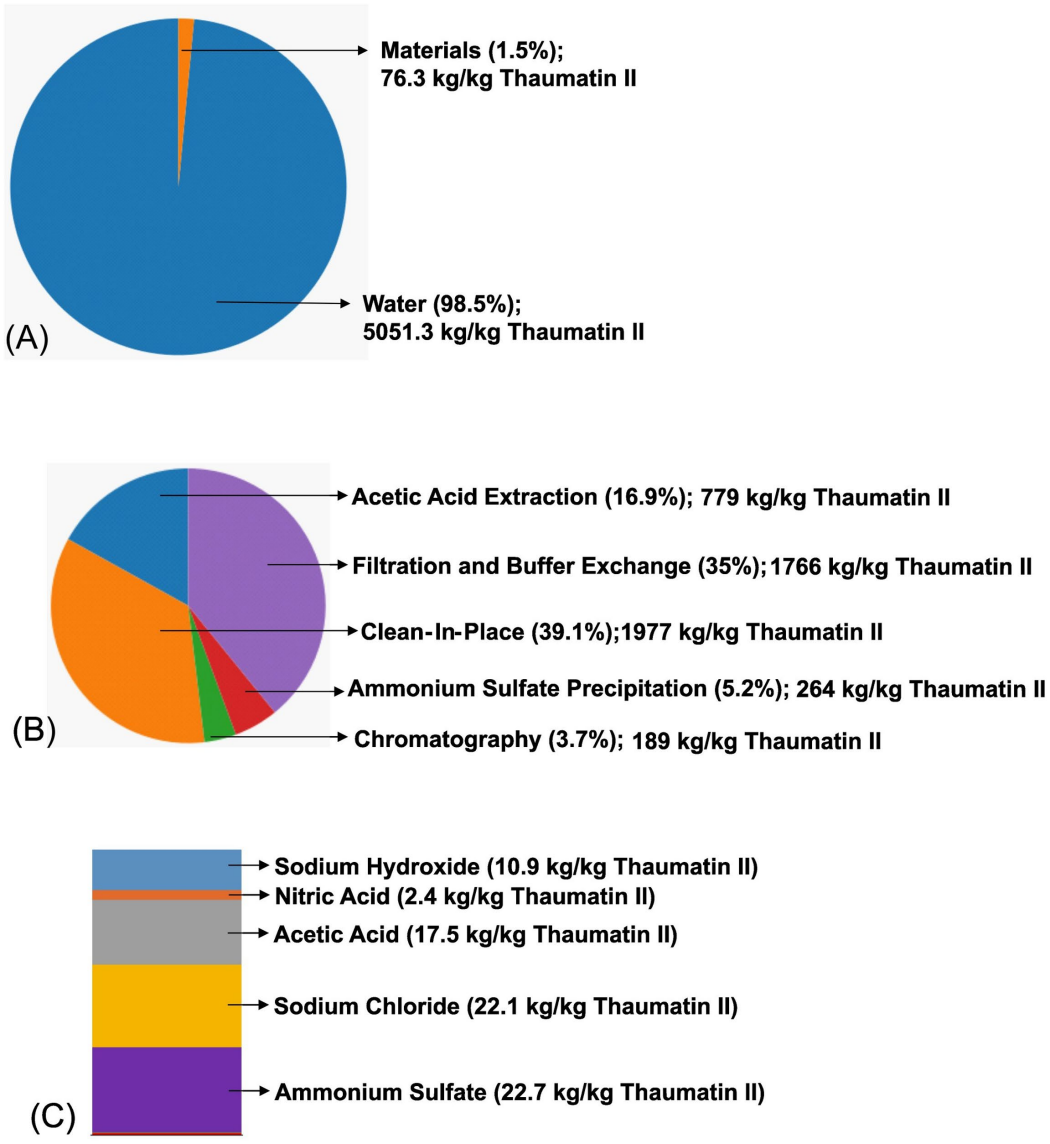
**(A)** Process mass intensity breakdown (material usage per kg Thaumatin II produced): 98.5% of the process mass is attributed to water; **(B)** Water consumption breakdown shows the water used during individual unit processes: Multiple filtration and buffer exchange steps associated with the high-salt extraction process are the dominant contributors to overall water use. CIP operations and acetic acid extraction also represent significant water-intensive stages, while chromatography and ammonium sulfate precipitation contribute comparatively smaller fractions to total water consumption; **(C)** Proportion of materials used in the process per kg of Thaumatin II produced.

Figure 5A illustrates that over 98.5% of the PMI is attributed to water consumption within the downstream purification process. A significant portion of this water is utilized during the multiple diafiltration steps for buffer exchange and salt removal, driven by the large volumes required for the high-salt extraction steps as seen in Figure 5B. Other major contributors to water usage include the acetic acid extraction process and the clean-in-place (CIP) procedures. Figure 5C shows the breakdown of all other materials used during the process. The PMI is used to calculate the environmental impact of the process.

### 3.4 Environmental impact analysis

**Figure 6:**
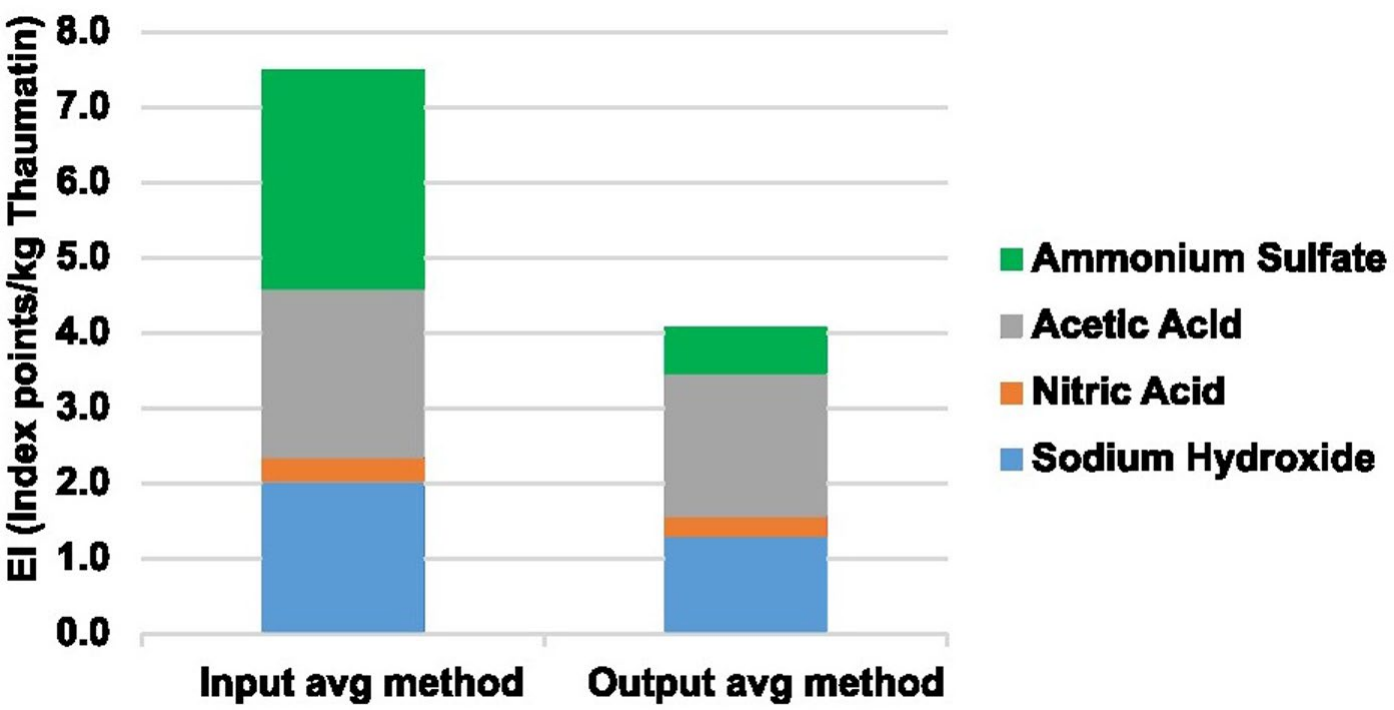
Environmental Index (EI) using the average method (24). Materials with an EI value of less than 0.05 are excluded from the figure.

The Environmental Index values for the downstream portion of the manufacturing process are calculated using the average method (Mv) (A = 1, B = 0.3, C = 0) (24) and are shown in Figure 6. For inputs, the impact groups include Resources, which accounts for raw material availability; Gray Input, reflecting the complexity of synthesis and the use of critical materials; Component Risk, associated with thermal risks; Organisms, which covers acute toxicity, chronic toxicity, and endocrine disruption potential. For outputs, the impact groups include Component Risk, which is associated with thermal risks; Organisms, covering acute toxicity, chronic toxicity, and endocrine disruption potential; Air, which encompasses global warming potential, ozone depletion potential, acidification potential, photochemical ozone creation potential, and odor; and Water/Soil, which includes eutrophication potential and organic carbon pollution potential by. These impact groups collectively form the Impact Group Indices (IGI), which indicate the contribution of each group to the overall environmental burden of the process. In this process, the input materials exhibit notable impacts related to thermal risk due to sodium hydroxide, acetic acid, and ammonium sulfate and Gray Input due to acetic acid, while the largest impact groups for the output streams are associated with Air, Water, and Soil due to sodium hydroxide, sodium hydrogen phosphate, and sodium dihydrogen phosphate.

The General Effect Index (GEI) is defined as the ratio of the EI to the MI and represents a weighted aggregate of the environmental impacts associated with all process inputs and outputs. The calculated GEI values, together with the corresponding Environmental Index values for the overall process, are presented in Table 5. Because the inclusion of water can disproportionately influence index-based sustainability metrics due to its large mass contribution, EI and GEI values were calculated both including and excluding water to better assess its effect on the overall environmental evaluation. The GEI (using the Mv method) ranges from 0 to 1, with lower values indicating improved environmental performance.

**Table 5:** Environmental performance of downstream purification process.

Parameter	Inputs	Outputs
EI (Mv) (Index point/kg Thaumatin II)	7.5	4.1
MI (kg/kg Thaumatin II)	76.3	76.3
GEI (Mv) including water (dimensionless)	0.0015	0.0008
GEI (Mv) excluding water (dimensionless)	0.16	0.21

GEI (Mv) metrics range from 0–1, with smaller numbers indicating lower environmental impact. EI, Environmental Index; MI, Mass Index; GEI, General Effect Index; Mv, Denotes use of the arithmetic average method in the calculation.

The GEI (Mv) values including water, 0.0015 and 0.0008, indicate a low overall environmental burden for the proposed process. Even with the contribution of water excluded, the GEI values are small. These low values are primarily attributable to the dominance of water in the Mass Index, combined with the relatively small environmental impacts of the remaining components. On the input side, sodium hydroxide is the only substance classified as Class A with respect to acute toxicity. However, because it is utilized at dilute concentrations, its contribution to the overall Environmental Index is limited. On the output side, only sodium hydrogen phosphate and sodium dihydrogen phosphate are classified as Class A for eutrophication potential. Although dilution does not mitigate eutrophication potential per se, these compounds constitute only 0.26 and 0.65% of the total process mass (excluding water), respectively, thereby resulting in a minimal overall contribution to the environmental impact.

The CO₂-equivalent emissions associated with electricity consumption in the process vary depending on the energy source. Emissions were estimated at 17.3 kg CO₂-eq per kg Thaumatin II when electricity was generated from coal, 7.2 kg CO₂-eq per kg Thaumatin II from natural gas, and 18.4 kg CO₂-eq per kg Thaumatin II from petroleum. When averaged across all energy sources, the emissions were 6.1 kg CO₂-eq per kg Thaumatin II. It should be emphasized that the values reported here account only for emissions associated with electricity consumption within the facility and do not represent a full life-cycle assessment.

## 4 Discussion

Techno-economic models allow rapid evaluation of the economics and profitability of biomanufacturing processes, such as Thaumatin II produced from tobacco seeds, including impacts of raw material costs, production level, and alternative downstream processing strategies. Although the results of this techno-economic analysis indicate that Thaumatin II produced in the designed facility is more expensive to produce on a direct mass basis than sucrose and perhaps other sweeteners, this comparison alone does not adequately capture the economic advantages of the seed-based process with respect to product usage in food formulations. Thaumatin II exhibits an exceptionally high sweetness intensity especially when blended with other sweeteners, enabling reductions in total sweetener usage. So, a better comparison would be in terms of Thaumatin II’s functional (sweetness) replacement potential. Sensory evaluation studies demonstrate that a solution containing 5% (w/v) sucrose supplemented with 4.6 ppm Thaumatin II achieves a sweetness equivalent to that of a 10% (w/v) sucrose solution (18). This corresponds to approximately 4.6 mg of Thaumatin II providing the same sweetness as 50 g of sucrose. At partial substitution levels, Thaumatin II enables large reductions in sucrose content while maintaining sweetness. When used as a 35–50% replacement for sucrose, the effective sweetness intensity exceeds 10,000 times that of sucrose on a mass basis.

Using market data from the USDA Sugar and Sweeteners Yearbook (29), the 2025 price of refined cane sugar is approximately $1.2/kg (dry basis), while high-fructose corn syrup is priced at $0.8/kg (dry basis). Based on the modeled minimum MSP of Thaumatin II of $814/kg (corresponding to a 10% internal rate of return with seed costs at $2/kg), a 50% replacement of refined sugar with Thaumatin II results in an estimated 47% reduction in sweetener cost on an equivalent sweetness basis. Even at a higher Thaumatin II price of $2,000/kg, the cost reduction remains substantial at approximately 42%. Similar trends are observed when 50% high-fructose corn syrup is replaced with Thaumatin II, where cost reductions are approximately 45% at $814/kg and 38.5% at $2,000/kg. These results demonstrate that, despite its higher production cost per mass relative to conventional sweeteners, the seed-based process analyzed is economically advantageous due to Thaumatin II’s high sweetness intensity. Thus, recombinant Thaumatin II offers a compelling cost advantage, particularly in reduced-sugar formulations targeting improved nutritional profiles.

The efficiency of the process described could be further improved upstream and downstream with the goal of increasing annual output (scalability) and reducing sweetener COGS (market competitiveness). Upstream (agronomic) improvements could include using tobacco varieties with higher seed yields; using growth regulators to induce flowering thus increasing the number of seed pods per plant from the current 250–300 (30); increasing the expression level of Thaumatin II in the seeds from the current 8 g/kg; planting at higher densities than those currently used for leaf tobacco (e.g., from 6,000–7,500 plants/acre (30) to >130,000 plants/acre (14)); and mechanization of seed pod harvesting to allow multiple harvests per season with greater efficiency. Although tobacco lends itself to facile genetic manipulation and hence was the focus of this initial study, crops other than tobacco that have higher yields of seeds per cultivated area, including food crops and forage crops (e.g., cereals including oats, barley, and rye), could also be considered for agronomic improvement once the molecular genetic tools for these species are optimized.

Similarly, downstream process improvements can be contemplated because the proposed process has a recovery of only 53.4%, indicating substantial product loss during purification. Significant amounts of Thaumatin II are lost during filtration, buffer exchange, and concentration operations. Process optimization could substantially enhance recovery. For example, implementing membrane filters with higher selectivity (improved rejection coefficients) for the target protein, while permitting host cell proteins and smaller impurities to pass during ultrafiltration and diafiltration, may reduce product losses and improve yield. Careful selection of membrane molecular weight cut-off and operating conditions would be critical to achieving this balance. An additional strategy for improving process performance is the use of evaporation as a pre-concentration step prior to spray drying. This approach could potentially reduce product loss associated with membrane-based concentration. However, thermal evaporation introduces trade-offs, including increased cost due to energy demand and associated CO₂ emissions. Future work can include a techno-economic and environmental assessment to determine whether the gains in recovery outweigh the added operational and sustainability costs.

An alternative approach to lowering COGS during downstream processing would be to lower the purity of Thaumatin II from the modeled 95%, as less stringent purification could result in lower product cost. Thaumatin II has been produced and purified from the biomass of various plant species to yield compositions whose impurities do not pose a safety concern, such as in the edible crop species *Beta vulgaris* (beet), *Spinacia oleracea* (spinach), or *Lactuca sativa* (lettuce) (16). Similarly, a recombinant version of the sweet protein brazzein produced by yeast fermentation has a final sweetener content of about 35% (w/w brazzein protein/final product). The product is categorized as FDA GRAS since neither the sweetener nor the impurities from the yeast host pose a safety concern (31). In the current analysis, tobacco seed could be included in this category because, unlike the leaf biomass of tobacco, the seeds are devoid of toxic alkaloids and are in fact safe and approved for use as a feed supplement (32). Hence, one could envision a process where the purity of Thaumatin II is less than 95% (w/w TSP), as long as the impurities do not interfere with the sweetness potency, functional properties, or the safety of the intended food or beverage product to which the sweetener is added (see purification Scenario 1 versus Scenario 2 in Section 3.1.2 and Table 4 for comparison).

It should be noted that TEA models have inherent uncertainties associated with process and economic parameters that were not considered in this study. Future work that integrates input parameter probability distributions into the model can provide the associated probability distributions of outputs such as COGS and MSP (15).

While the preceding economic analysis demonstrates that even with the current parameters the production of Thaumatin II in tobacco seeds is commercially viable, the advantages of the recombinant production process extend beyond economics. The production of sugar substitutes using recombinant processes, such as the one described in this study, could also offer substantial environmental benefits compared to the manufacture of conventional carbohydrate sweeteners. Relative to sugar production, these benefits could include lower land use, reduced greenhouse gas emissions, significantly lower irrigation water demand, and improved conservation of soil quality. Although our study focused on downstream process efficiencies and not on upstream agronomic practices, some of the advantages of the overall process are worth mentioning. For example, our initial pilot-scale tobacco field studies yielded >8 kg/hectare (~3.3 kg/acre) of purified Thaumatin II (data not shown), which translates to a sweetness equivalent to >80 MT of sugar per hectare (~33 MT/acre). That is, with sugarcane and sugar beets averaging yields of 7–10 MT of obtainable sucrose per hectare (~2.8–4 MT/acre; (33)), the sweetness productivity difference of Thaumatin II is ~10-times higher than that of sucrose on an agricultural area basis.

Similarly, according to the Food and Agriculture Organization of the United Nations (34), the evapotranspiration maximum (ETm), defined as the estimated total maximum water requirement for plant growth plus soil evaporation and plant transpiration during a crop’s cultivation cycle, ranges from 1,500 to 2,500 mm for sugarcane and from 400 to 600 mm for tobacco (ranges are cited due to annual differences due to location and weather conditions). That is, about 4 times more water is needed to cultivate a given area of sugarcane than that required for producing tobacco. Post-harvest processing and washing of sugarcane requires from 3 to 10 MT water per MT of cane, followed by water usage in boilers to extract and concentrate the sugar prior to evaporation and crystallization, equipment cleaning, etc. (4). In all, downstream extraction/concentration of sugarcane-derived sucrose requires from 1.5 to 3 kL water/kg sugar (4). Although the downstream water requirement for Thaumatin II in the modeled facility on a mass basis is higher than that reported for sugarcane-derived sucrose (~5 kL/kg Thaumatin II vs. 1.5–3 kL/kg sucrose), Thaumatin II is >10,000-times sweeter than an equivalent weight of sucrose; therefore the water conversion efficiency to produce equivalent sweetness decidedly favors cultivation of Thaumatin II.

In addition to depleting regional water for other uses, sugarcane production is associated with deforestation, eutrophication, soil erosion and loss of soil quality, especially in subtropical and tropical environments (4) whereas tobacco can be cultivated in marginal land where soil quality is much less of a concern. Life cycle assessment studies on the production of thaumatin by extraction (10) and of other natural high-intensity sweeteners highlight the magnitude of additional environmental advantages. For example, the production of steviol glycosides extracted from stevia cultivated in Europe has been shown to generate only approximately 10% of the greenhouse gas emissions associated with sugar production (35). While environmental and sustainability benefits may extend broadly to natural sweeteners, production of Thaumatin II is particularly compelling due to the protein’s exceptional sweetness potency.

With respect to thaumatin specifically, Suckling et al. (10) conducted a life cycle assessment of thaumatin produced from the arils of *Thaumatococcus daniellii* fruit harvested from forests in West Africa and processed in the United Kingdom. Environmental impact was reported for many of the same categories used in our analysis, including global warming potential, land use, water consumption, and water eutrophication. Impacts were expressed in terms of product mass and sweetness potency or equivalence. Global warming potential for production of thaumatin from fruit was found to be 719.2 kg CO_2_-eq/kg. Of this total, the study attributed 63.8 kWh of electricity consumption to the extraction process, corresponding to 23.6 CO₂-eq per kg thaumatin (assuming a natural gas-based electricity source). This indicates a higher carbon intensity compared to the electricity-related emissions of the present process (7.2 CO₂-eq per kg Thaumatin II, see Section 3.4); however, the values reported here are for emissions associated with electricity consumption within the facility and do not represent a full life-cycle assessment. When fruit-extracted thaumatin replaces 20% of added sugar, environmental impact for a given sweetness was found to be 19.4% lower across all impact categories. International transport under cold storage of the arils (from West Africa to the EU and UK) was found to be a major contributor to global warming potential, as was aril removal from the fruit to freshwater eutrophication and water use (10).

For comparison, the recombinant Thaumatin II production process modeled here begins with cultivated source-plant material (tobacco seed), not foraged fruit, which in the traditional process accounts for most of the impact on land use (10). Unlike harvested fruit, recombinant seeds containing Thaumatin II do not need cold storage for preservation or transport, and extraction of the sweetener from the seeds can be done in one or more modular facilities located in or in proximity to tobacco-growing regions. This would obviate the need for long-distance transport of materials, with expected reductions in environmental impact. Additional comparisons of the seed-based process to the traditional fruit extraction method were beyond the scope of this study but could be addressed in a future evaluation.

As described herein, the most economic method of producing Thaumatin II is by expression of the protein in the seeds of tobacco plants (*Nicotiana tabacum*). Unlike tobacco’s green biomass, the seeds of the tobacco varieties used to produce the sweetener contain no nicotine or other pyridine alkaloids. Because the seeds represent a very small fraction of the total tobacco biomass, extraction of Thaumatin II from seeds can be achieved using smaller equipment housed in facilities with a smaller footprint, thereby reducing capital and operating costs. Additionally, production of Thaumatin II in the seeds leaves behind residual biomass that can be valorized. The remaining biomass has potential applications as a source of co-products, including seed oil, leaf nicotine, and dry matter for energy generation, or animal feed. Although not quantified in this study, there is also the possibility of using the residual tobacco leafy biomass for production of biofuels (36), natural products (37), or valuable recombinant proteins (38). In addition, the defatted tobacco seed cake can be used as an animal feed and has been approved for such use in the European Union since 2010, per Regulation EC 767/2009 (32), including cakes from high-oil content seeds used for biofuel generation (39). The expected energy yields of this residual biomass are comparable to those of established biomass crops currently cultivated for bioenergy production (40). Since substantially smaller quantities are required to achieve equivalent sweetness compared to sucrose or other sweeteners, the environmental benefits per unit of delivered sweetness are expected to be even more pronounced. Such analyses could be the subject of a subsequent study.

Beyond its economic and environmental advantages, Thaumatin II has the potential to advance global public health objectives. Low- and reduced-calorie sweeteners enable substantial reductions in added sugar intake while maintaining sweetness, a critical determinant of consumer acceptance. Such sugar substitutes play a central role in global strategies to mitigate unhealthy weight gain and reduce the prevalence of diet-related chronic diseases, including obesity, cardiovascular disease, and dental caries. Policy interventions, such as sugar taxes implemented in multiple countries, have yielded encouraging outcomes. In the United Kingdom, where sugar levies have been in effect since 2018, these measures are estimated to have prevented more than 5,000 cases of obesity annually among certain child populations (41). Because reduced sweetness is generally unacceptable to consumers, these policies have accelerated the adoption of products in which sugar is partially replaced with low- and no-calorie sweeteners (LNCS).

LNCS consumption also offers benefits for individuals with diabetes. Meta-analyses report improvements in body weight and cardiometabolic risk factors when LNCS-sweetened beverages replace sugar-sweetened alternatives, without evidence of harm (42). Similar supporting studies have led to new diabetes management guidelines to include water or LNSC as replacements for caloric sweeteners (2). Thaumatin II, with a zero glycemic index and no effect on blood glucose levels (43), is well suited for this role. Its widespread adoption could enable up to a 50% reduction in dietary sugar while potentially lowering overall LNCS usage through high-potency sweetener blends.

## 5 Conclusion

While the market benchmark price for the commercial extract (Talin®) is $7,000/kg, the recombinant process achieved a unit production cost of $766/kg for Thaumatin II of 95% purity (Thaumatin II/total soluble protein) and 5% moisture content, in a modeled facility capable of producing 50 metric tons of Thaumatin II annually. This study shows that seed-based recombinant production of Thaumatin II offers a viable alternative to conventional carbohydrate sweeteners when evaluated across economic, environmental, and public health dimensions. Despite higher production costs on a mass basis, Thaumatin II’s exceptional sweetness intensity enables significant reductions in total sweetener use, making it economically competitive on a cost-per-sweetness basis. The production process also provides notable environmental benefits, including reduced land and water use, lower greenhouse gas emissions, and opportunities for residual biomass valorization. Importantly, Thaumatin II can support global public health objectives by enabling substantial reductions in added sugar intake without compromising sweetness. With a zero glycemic index and suitability for individuals with diabetes, Thaumatin II represents a promising ingredient for advancing healthier and more sustainable food systems. Collectively, these factors further strengthen the argument of Thaumatin II as an economic, sustainable, and healthy alternative to conventional caloric sweeteners. Future work should focus on improving upstream specific yield of Thaumatin II in tobacco or other crops and downstream recovery to enhance overall process efficiency and product yield. It is important also to evaluate the associated economic and environmental impacts of such improvements to ensure a more efficient, cost-effective, and sustainable manufacturing process.

## Data Availability

The original contributions presented in the study are included in the article/Supplementary material, further inquiries can be directed to the corresponding author. In addition, the process modeling base-case file generated in this study can be accessed at https://mcdonald-nandi.ech.ucdavis.edu/tools/techno-economics/. A free trial download of SuperPro Designer modeling software can be accessed at https://www.intelligen.com/download.
