# Supplementary material for: Techno-economic assessment for plant seed-based production of Thaumatin II: a natural high intensity protein sweetener
Source: Front Nutr. 2026 Jul 23;13:1868916. doi: 10.3389/fnut.2026.1868916 (PMC13441740; doi:10.3389/fnut.2026.1868916)
Supplement: Supplementary file 1 [file Table_1.docx]

*Supplementary Material*

**Techno-Economic Assessment for Plant Seed-Based Production of Thaumatin II: a Natural High Intensity Protein Sweetener**

Varun Gore^1^, Daniel Tusé^2^, Anatoli Giritch^3^, Somen Nandi^1, 4^, and Karen A. McDonald^1, 4*^

^1^ Department of Chemical Engineering, University of California, Davis, California, United States

^2^ DT/Consulting Group, Sacramento, California, United States

^3^ Nomad Bioscience GmbH, Halle (Saale), Germany

^4^ Global HealthShare^®^ Initiative, University of California, Davis, California, United States

* Corresponding author: [kamcdonald@ucdavis.edu](mailto:kamcdonald@ucdavis.edu)

Table S1: Downstream processing losses breakdown per unit operation. All loss values are obtained from pilot scale experimental data.

| Step | Loss (% of initial Thaumatin II) | Cumulative recovery (% of initial Thaumatin II) |
| --- | --- | --- |
| Grinding | 5 | 95 |
| Acetic Acid extraction | 5.6 | 89.4 |
| Buffer exchange and concentration (10kDa) | 24 | 65.4 |
| Chromatography | 7.5 | 57.9 |
| Ammonium Sulfate precipitation | 0.5 | 57.4 |
| Buffer exchange and concentration (5kDa) | 4 | 53.4 |
| Drying | 0 | 53.4 |

Table S2: Downstream purification facility Direct Fixed Capital (DFC) estimation parameters. Values are not directly reported in the cited references and were derived through interpolation based on information for a mixed fluid-solid processing plant (1) and a food processing plant (2). All values are author assumptions based on intermediate values between the two reported facility types.

| Parameter | Factor |
| --- | --- |
| **Direct Costs (DC)** |  |
| Equipment Purchasing Cost (PC)  Values derived from SuperPro Designer® default values | 1 x PC |
|  |  |
| Unlisted Equipment | 0.20 x PC |
|  |  |
| **Direct Costs (DC)** |  |
| Piping (A) | 0.20 x PC |
| Instrumentation (B) | 0.20 x PC |
| Insulation (C) | 0.01 x PC |
| Electrical Facilities (D) | 0.05 x PC |
| Buildings (E) | 0.23 x PC |
| Yard Improvement (F) | 0.08 x PC |
| Auxiliary Facilities (G) | 0.20 x PC |
|  |  |
| Installation | 0.15 x PC |
|  |  |
| DC = PC + unlisted equipment + Installation + A + B + C + D + E + F + G | |
| **DC = 2.12 PC** |  |
|  |  |
| **Indirect Costs (IC)** |  |
| Engineering | 0.44 x PC |
| Construction | 0.44 x PC |
|  |  |
| **IC = 0.88 PC** |  |
|  |  |
| Other Costs (OC) |  |
| Contractor's Fee | 0.15 x PC |
| Contingency | 0.30 x PC |
|  |  |
| **DFC = DC + IC + OC** | 3.45 x PC  **(Lang Factor)** |

Table S3: Working capital (WC) estimation parameters for downstream processing facilities. All values are SuperPro Designer default values.

| Parameter | Value |
| --- | --- |
| Cover labor expenses for | 30 days |
| Cover raw materials expenses for | 30 days |
| Cover utilities expenses for | 30 days |
| Cover waste treatment expenses for | 30 days |
| Startup and Validation | 5% of DFC |

Table S4: Downstream Purification facility operating cost factors. All values are SuperPro Designer® default values.

| Parameter | Cost factor | |
| --- | --- | --- |
| **Facility dependent costs** |  | |
| Maintenance | Equipment specific (SuperPro Designer® default values) | |
| Depreciation | 10-year straight line (5% salvage value) | |
| Insurance | 1% DFC | |
| Local Taxes | 2% DFC | |
| Factory expense | 5% DFC | |
|  |  | |
| **Labor** |  |  |
| Type | basic rate | lumped rate |
| Downstream operator | 29.9 USD/h | 59.8 USD/h |
|  |  | |
| **Utilities** |  | |
| Type | Unit cost | |
| Standard Power | 0.1 USD/ kWh | |
| Steam | 12 USD/MT | |
| Chilled Water | 0.4 USD/MT | |

References

1.	Peters MS, Timmerhaus KD, West RE, West RE. *Plant design and economics for chemical engineers*. 5. ed, international ed. 2004. Boston: McGraw-Hill. (2004). 988 p.

2.	Marouli AZ, Maroulis ZB. Cost data analysis for the food industry. *J Food Eng* (2005) 67:289–299. doi: 10.1016/j.jfoodeng.2004.04.031
